# Associated factors of safe child feces disposal in sub-Saharan Africa: Evidence from recent demographic and health surveys of 34 sub-Saharan countries

**DOI:** 10.1371/journal.pone.0281451

**Published:** 2023-02-09

**Authors:** Getu Debalkie Demissie, Muluken Fekadie Zerihun, Daniale Tekelia Ekubagewargies, Yigizie Yeshaw, Tadeg Jemere, Bisrat Misganaw, Amare Tariku, Asmamaw Atnafu

**Affiliations:** 1 Department of Health Education and Behavioral Sciences, Institute of Public Health, College of Medicine and Health Sciences, University of Gondar, Gondar, Ethiopia; 2 Department of Biochemistry, School of Medicine, College of Medicine and Health Sciences, University of Gondar, Gondar, Ethiopia; 3 Department of Pediatrics and Child Health Nursing, School of Nursing, College of Medicine and Health Sciences, University of Gondar, Gondar, Ethiopia; 4 Department of Human Physiology, School of Medicine, College of Medicine and Health Sciences, University of Gondar, Gondar, Ethiopia; 5 Department of Epidemiology and Biostatistics, Institute of Public Health, College of Medicine and Health Sciences, University of Gondar, Gondar, Ethiopia; 6 Department of Biomedical Sciences, College of Health Sciences, Debretabor University, Debretabor, Ethiopia; 7 Department of Human Nutrition, Institute of Public Health, College of Medicine and Health Sciences, University of Gondar, Gondar, Ethiopia; 8 Department of Health Systems and Policy, Institute of Public Health, College of Medicine and Health Sciences, University of Gondar, Gondar, Ethiopia; Madda Walabu University, ETHIOPIA

## Abstract

**Introduction:**

Children’s feces are thought to pose a greater public health risk than those of adults’ due to higher concentrations of pathogens. The aim of this study was to determine the associated factors of safe child feces disposal among children under two years of age in Sub-Saharan Africa.

**Methods:**

The most recent demographic and health survey datasets of 34 sub-Saharan countries were used. A total weighted sample of 78, 151 mothers/caregivers of under two children were included in the study. Both bivariable and multivariable multilevel logistic regression were done. The Odds Ratio (OR) with a 95% Confidence Interval (CI) was calculated for each independent variables included in the model.

**Results:**

Those mothers/caregivers from urban residence (AOR = 1.42; CI: 1.36, 1.48), mothers with primary education (AOR = 1.49; CI: 1.44, 1.56), richer (AOR = 1.78; CI: 1.69, 1.88) and richest wealth quintiles (AOR = 2.17; CI: 2.01, 2.31), family size <5 (AOR = 1.06; CI: 1.02–1.09), access to improved water source (AOR = 1.29; CI: 1.25, 1.34), mothers who owned toilet (AOR = 3.09; 2.99–3.19) and who had media exposure (AOR = 1.19; CI: 1.15, 1.24) had higher odds of practicing safe child feces disposal than their counter parts. However, mothers/care givers who are not currently working (AOR = 0.83; CI: 0.80, 0.86), higher education (AOR = 0.85; CI: 0.76–0.94) and from Western region of Africa (AOR = 0.82; CI: 0.79–0.86) had reduced chance of safe child feces disposal as compared to their counter parts.

**Conclusion:**

Residence, mothers’ level of education, wealth index, water source, toilet ownership and media exposure were factors associated with safe child feces disposal. It is advisable to implement health promotion and behavioral change intervention measures especially for those women /caregivers from rural residence, poor economic status, who cannot access improved water and for those with no media exposure to improve the practice of safe child feces disposal.

## Introduction

To end open defecation, access to adequate sanitation and hygiene for all facilities is still an issue and a cross cutting problem throughout the globe [1]. Despite the practice of open defecation is common among children in many low-income countries, so far little attention is given for the problem in the region [2, 3].

Open defecation accelerates the transmission of gastro intestinal diseases [4, 5]. This problem becomes more pronounced in children because of their feces are thought to pose a greater public health risk (child’s fecal contains higher concentrations of pathogens) than those of adults’. Furthermore, parent’s perceive that infants’ feces are harmless and they are often allowed to practice open defecation in the household [6, 7]. This causes an increased risk of getting diarrhea and others infectious diseases among young children [8–10].

Unsafe child feces disposal practices increased the risk of diarrheal diseases by 23% [10]. Moreover, it also affects the growth of children [11–13]. On the other hand, safe child feces disposal resulted in a 35% reduction in helminths infections [2]. Different studies also found that improving water supply and hygiene practices including safe disposal of children’s feces, could prevent 361,000 deaths per a year in children below five [14, 15].

The finding of previous studies revealed that wealth [14, 16–18], media exposure [14, 16, 19], place of residence [14, 18, 20], latrine facility [17, 19, 21–23], mothers’ age [18, 20–23], source of drinking water [14, 23], child’s age [18, 19, 23], education level of mothers/care givers [17, 19], number of children <5 years of age in the household [17] and occupational status of mothers [20] were associated with child feces disposal.

Despite there are related studies in some sub-Saharan African countries, there is very limited evidence on the pooled estimate and associated factors of safe child feces disposal across the region. Hence, this study was conducted to determine the associated factors of safe child feces disposal among children under two years of age in SSA. As this study used the pooled DHS of 34 Sub Saharan countries, it helps increase the power of the study.

## Methods

### Data source and data quality control

This study was a secondary data analysis from the most recent appended demographic and health surveys (KR data sets) conducted in 34 sub-Saharan countries from 2009 to 2018. The DHS is a nationally representative survey that collects data on basic health indicators like morbidity, mortality, fertility, maternal and child health. The DHS used two stage stratified sampling technique to select the study participants.

A pretested and standard DHS questionnaires were used for data collection of the DHS surveys. The questionnaire was conceptualized to the different countries context and the data were collected by trained data collectors. The datasets of each sub- Saharan country were obtained at https://dhsprogram.com/data/dataset_admin/index.cfm.Those countries which have data on feces disposal among under two children were included in the analysis. We removed those cases which were incomplete from the analysis to handle missing data. Each country was given a code and then appended together to create a single data set that represents the SSA countries. In this study, a total weighted sample of 78,151 under-two children were included (Table 1).

**Table 1:** List of sub-Saharan countries and their demographic and health survey’s year included in the analysis.

Country name	Survey year
Angola	2015/16
Burkina Faso	2010
Burundi	2016/17
Cameroon	2018
Congo Democratic Congo	2013/14
Chad	2015
Comoros	2012
Congo	2011/12
Cote d’vore	2011/12
Ethiopia	2016
Gabon	2012
Ghana	2014
Gambia	2013
Guinea	2018
Kenya	2014
Liberia	2013
Lesotho	2014
Madagascar	2009
Mali	2018
Malawi	2015/16
Mozambique	2011
Nigeria	2018
Niger	2012
Namibia	2013
Rwanda	2014/15
Sera lone	2016
Sao tome and Principe	2009
Senegal	2011
Togo	2013
Tanzania	2015/16
Uganda	2016
South Africa	2016
Zambia	2018
Zimbabwe	2015

### Variables of the study

#### Dependent variable

The dependent variable for this study was safe child feces disposal. Safe child feces disposal is a binary outcome (yes or no) and a child is said to have safe child feces disposal if they used latrine’ and if they ‘put/rinsed child feces into latrine [18].

#### Independent variables

The independent variables considered for this study were both individual and community level variables. The individual level variables were age of child, age of mothers, education level of mothers, education level of partners, wealth index, occupational status of mothers, family size, number of under five children, water source, toilet ownership and media exposure (a composite variable generated by the aggregation of listening radio, reading newspaper and watching television and it was dichotomized as yes “if the mother had exposure to either of the above three mentioned media sources” and no “if she had no exposure to all of the three media sources). SSA region and residence were considered as the community level variables.

#### Data analysis procedure

Data extraction, recoding and analysis were done using STATA version 14 software. Before the analysis sampling weight was applied to produce reliable estimates by adjusting the over and under-sampled region. Sample weights were calculated to six decimals but are presented in the standard recode files without the decimal point. They need to be divided by 1,000,000 before use to approximate the number of cases. The whole procedure of weighting and its rationale is found on the guide of DHS statistics [24].

Measures of community variation/random effects such as Median Odds Ratio (MOR), Proportional Change in Variance (PCV) and Interclass Correlation Coefficient (ICC) were calculated due to the correlated nature of DHS data. Accordingly, the values of these measures were found to be significant and hence the use of multilevel logistic regression model is more appropriate than ordinary logistic regression. To choose the best fitted model, first we developed four models and compared them with deviance. The first one is the null-model; a model with no independent variable, the second model is model I; a model that has individual-level factors only, model II; a model with community-level factors only and model III; a model that contain both individual and community level independent variables. Of the four models fitted, model III was selected as the best fitted model (it had the lowest deviance).

Then after, both bivariable and multivariable multilevel logistic regression was conducted to determine the associated factors of safe child feces disposal in SSA. All variables with a p value < 0.2 at bi-variable analysis were entered into the multivariable logistic regression model. In the final model p value ≤ 0.05 was used to declare statistically significant variables.

## Ethical consideration

We have received a permission letter to download and use the data files from DHS Program.

## Results

### Sociodemographic characteristics of the respondents

The total weighted samples of 78, 151 under-two children were included in this study. Seventy two percent of children were from the rural area. Nighty eight percent of children (97.9%) were in the age group of 0–11 months. Of their mothers/caregivers, 46.3% were in the age group of 25–34 years, 39.8% had no education. Majority of their mothers/caregivers had media exposure (62.6%) and used improved water source (63.0%). (Table 2)

**Table 2:** Sociodemographic characteristics of the respondents in sub-Saharan Africa, 2020.

Variable	Category	Weighted frequency	Percent (%)
Age of child (in months)	0–11	76,557	97.9
	12–23	1,595	2.1
Residence	Urban	21,831	27.9
	Rural	56,320	72.1
SSA region	East Africa	17,570	22.5
	West Africa	26,332	33.7
	South Africa	12,879	16.5
	Central Africa	21,370	27.3
Age of mothers(years)	15–24	27,564	35.3
	25–34	36,185	46.3
	35 and above	14,402	18.4
Education level of mothers	No formal education	31,078	39.8
	Primary	26,776	34.3
	Secondary	17,811	22.8
	Higher	2,486	3.1
Education level of partners	No formal education	34,262	43.8
	Primary	21,195	27.1
	Secondary	18,285	23.4
	Higher	4,409	5.7
Working status of mothers	Currently working	48,057	61.5
	Currently	30,094	38.5
	Not working		
Family size	<5	32,677	41.8
	≥5	45,474	58.2
Number of under 5 children in the house hold	≤2	56,396	72.2
	3–4	18,412	23.6
	≥5	3,343	4.2
Wealth index	Poorest	17,879	22.9
	Poorer	16,868	21.6
	Middle	15,769	20.2
	Richer	14,830	18.9
	Richest	12,803	16.4
Water source	Improved	49,217	63.0
	Un improved	28,934	37.0
Toilet ownership	Yes	44,901	57.5
	No	33,250	42.5
Media exposure of mothers	Yes	48,683	62.3
	No	29,468	37.7

### Random effect analysis

The random-effects model result showed that there is significant clustering of practicing safe child feces disposal across the communities (OR of community level variance = 0.53, 95% CI = 0.47–0.59). The value of ICC in the null model revealed that 13.88% of the overall variation of practicing safe child feces disposal was attributed to cluster variability. The 1.99 MOR value of the null model also indicates the presence of variation to practice safe child feces disposal between clusters. It means if we randomly select households from different clusters, those households at the cluster with higher practicing safe child feces disposal had 2 times higher chance of practicing safe child feces disposal compared to their counterparts. As you can see in the Table 3 below, model III has the lowest Deviance value. Hence, it was selected as best fitted model (Table 3).

**Table 3:** Model comparison and random effect analysis result.

Parameters	Null model	Model I	Model II	Model III
Community level variance	0.53(0.47–0.59)	0.63(0.56–0.71)	0.49(0.43–0.55)	0.50(0.44–0.57)
ICC	13.88%	16.16%	12.92%	13.26%
MOR	1.99	2.08	1.94	1.96
PCV	Ref	18.87%	7.51%	5.66%
**Model fitness**				
Deviance	356,185.76	346,401.12	308,537.76	306,855.18

### Factors associated with safe child feces disposal

The odds of practicing safe child feces disposal among urban residents were 1.42 (AOR = 1.42; CI: 1.36, 1.48) times higher as compared to rural residents. The odds of practicing safe child disposal for mothers/care givers from Western Africa, Southern Africa and Central Africa were decreased by 18% (AOR = 0.82; CI: 0.79–0.86), 8% (AOR = 0.92; CI: 0.88–0.97) and 16% (AOR = 0.84; CI: 0.79–0.87) as compared to women from Eastern African respectively.

The odds of practicing safe child feces disposal among mothers/caregivers whose education level was primary and secondary were 1.49 (AOR = 1.49; CI: 1.44, 1.56) and 1.35 (AOR = 1.35; CI: 1.28, 1.41) times higher as compared to those who did not have formal education, respectively. However, the odds of practicing safe child feces disposal among mothers/care givers who had higher education level was decreased by 15% (AOR = 0.85; CI: 0.76–0.94) as compared to their counter parts.

The odds of practicing safe child feces disposal among mothers/care givers who are not currently working decreased by 17% (AOR = 0.83; CI: 0.80, 0.86) as compared to women who were currently working. Women/caregivers with family size <5 were 1.06(AOR = 1.06; CI: 1.02, 1.09) times more likely to practice safe child feces disposal as compared to their counter parts. Those children from poorer, middle, richer and richest households had 1.34 (AOR = 1.34; CI: 1.28, 1.41), 1.51 (AOR = 1.51; CI: 1.44, 1.48), 1.78 (AOR = 1.78; CI: 1.69, 1.88) and 2.17 (AOR = 2.17; CI: 2.01, 2.31) times higher odds of safe child feces disposal practice as compared to households from the poorest wealth quintiles, respectively.

Those households who can access improved water had 1.29 (AOR = 1.29; CI: 1.25, 1.34) times higher odds to practice safe child feces disposal as compared to their counter parts. Similarly, those mothers/caregivers who had not toilet had about 3.09 (AOR = 3.09; CI: 2.99, 3.19) times higher odds of practicing safe child feces disposal than their counter parts. Furthermore, mothers/caregivers who had media exposure were 1.19 (AOR = 1.19; CI: 1.15, 1.24) times more likely to practice safe child feces disposal as compared to their counter parts (Table 4).

**Table 4:** Multilevel regression analysis of safe child feces disposal among child <2 years in sub-Saharan Africa.

Variables	Safe child stool disposal		Odds ratio	
	Yes	No	COR (95%CI)	AOR (95%CI)
Residence				
Urban	14,015 (64.2)	7,815 (35.8)	2.50(2.45–2.55)	1.42(1.36–1.48)*
Rural	25,998 (46.2)	30,321 (53.8)	1	1
Age of child(in month)				
0–11	39,256 (51.3)	37,300 (48.7)	1	1
12–23	758 (47.6)	836 (52.4)	0.80(0.72–0.86)	0.96(.82–1.02)
Age of mothers (years)				
15–24	13,944 (50.6)	13,620 (49.4)	1	1
25–34	18,768 (51.8)	17,417 (48.2)	1.04(1.02–1.06)	1.02(0.98–1.05)
35 and above	7,302 (50.7)	7,098 (49.3)	0.99(0.97–1.01)	0.99(0.94–1.04)
Region				
East Africa	9,697 (55.2)	7,872 (44.8)	1	1
West Africa	12,784 (48.6)	13,548 (51.6)	0.87(0.85–0.0.88)	0.82(0.79–0.86) *
South Africa	7,373 (57.3)	5,505 (42.8)	1.09(1.06–1.12)	0.92(0.88–0.97) *
Central Africa	10,159 (47.5)	11,210 (52.5)	1.06(1.03–1.08)	0.84(0.79–0.87) *
Education level of mothers				
No education	12,515 (40.3)	18,563 (59.7)	1	1
Primary	15,154 (56.6)	11,622 (43.4)	2.08(2.04–2.12)	1.49(1.44–1.56) *
Secondary	10,824 (60.8)	6,987 (39.2)	2.56(2.50–2.62)	1.35(1.28–1.41) *
Higher	1,521 (61.2)	964 (38.8)	3.27(3.09–3.45)	0.85(0.76–0.94) *
Education level of Partner’s				
No education	15,007 (43.8)	19,254 (56.2)	1	1
Primary	11,615 (54.8)	9,579 (45.2)	1.74(1.70–1.77)	1.23(1.18–1.28) *
Secondary	10,571 (57.8)	7,714 (42.2)	1.92(1.88–1.96)	1.16(1.11–1.22) *
Higher	2,820 (63.9)	1,588 (36.1)	3.09(2.98–3.22)	1.09(1.01–1.18) *
Respondents’ occupations				
working	25,383 (52.8)	22,673 (47.2)	1	1
not working	14,631 (48.6)	15,463 (51.4)	0.81(0.79–0.82)	0.83(0.80–0.86) *
Number of under five children				
≤2	29,841(52.9)	26,555(47.1)	1.11(1.03–1.19)	1.06(0.98–1.15)
3–4	8,539(46.4)	9,873(53.6)	0.83(0.77–0.89)	0.87(0.88–0.95) *
≥5	1,634(48.9)	|1,708(51.1)	1	1
Family size				
<5	17,156 (52.5)	15,520 (47.5)	1.09(1.07–1.10)	1.06(1.02–1.09) *
≥5	22,857 (50.3)	22,616 (49.7)	1	1
Wealth index				
Poorest	6,324 (35.4)	11,554 (64.6)	1	1
Poorer	7,873 (46.7)	8,995 (53.3)	1.59(1.55–1.63)	1.34(1.28–1.41) *
Middle	8,274 (52.5)	7,494 (47.5)	1.99(1.95–2.06)	1.51(1.44–1.48) *
Richer	8,824 (59.5)	6,005 (40.5)	2.62(2.56–2.69)	1.78(1.69–1.88) *
Richest	8,717 (68.1)	4,086 (31.9)	5.22(5.08–5.38)	2.17(2.01–2.31) *
Water source				
Improved	27,965 (56.8)	21,251 (43.2)	1.89(1.87–1.93)	1.29(1.25–1.34) *
Un improved	12,048 (41.6)	16,885 (58.4)	1	1
Toilet ownership				
Yes	17,865 (39.8)	27,036 (60.2)	1	1
No	22,149 (66.6)	11,100 (33.4)	4.03(3.96–4.10)	3.09(2.99–3.19) *
Media exposure				
Yes	27,708 (56.9)	20,974 (43.1)	1.91(1.87–1.94)	1.19(1.15–1.24) *
No	12,305 (41.8)	17,162 (58.2)	1	1

* P<0.2 for COR and P≤0.05 for AOR

## Discussion

The aim of this study was to determine the associated factors of safe child feces disposal among under two children in sub-Saharan African countries. This study revealed that mothers/caregivers from urban residence had higher chance to practice safe child feces disposal compared to mothers from rural residence. This finding is similar with other studies conducted in Ethiopia [14, 18] and Kenya [25]. The possible reason could be residents from urban might have improved sanitation services than rural residents which in turn influence their hygienic behavior [26]. Moreover, mothers/ caregivers in an urban set up might have better access to health information [20].

This study showed that mothers/ caregivers and their partners with primary and secondary education level practice safe child feces disposal than non-educated ones. This finding was in line with a study conducted elsewhere [25, 27–29]. The possible reason might be educated mothers and partners can understand the causes of child illness and they are clearly aware of the deleterious effects of unsafe child feces disposal [30].

In this study, it was found that children from households with high wealth index had higher chance of practicing safe child waste disposal than the poorest one. This finding is consistent with other studies in Ethiopia [14, 18], India [17] and South Africa [16]. Based on the NFHS-3 report, stools were safely disposed for 65% of children in households with higher socioeconomic status, while only 4% of children in households with lower socioeconomic status [31]. This indicates that how wealth has a direct implication to access basic water and sanitation services which in turn influence on safe child feces disposal [32].

This study also revealed that mothers who were not employed were less likely to practice safe child feces disposal than mothers who were employed. This finding is inconsistent with other previous studies [7, 33]. This might be because of the reason that mothers who were not employed usually less educated and this might influence their hygienic behavior [30]. In agreement of other studies [17, 21, 34] household members less than five were more likely to practice safe child feces disposal. Having lower number of family size might reduce the workload of mothers/caregivers which makes them to have sufficient time to practice better hygienic behavior [35].

This study further revealed that media exposure was significantly associated with safe child feces disposal which is consistent with other studies in Cambodia [34] and India [28]. A similar previous study in Burkina Faso suggested that health promotion programs through health education and mass media, resulted in 4% increase in safe disposal of stool [36]. In agreement of other studies in Nigeria [37] and India [3], the ownership of toilet facility was associated with safe practice of child feces disposal. Possessing latrine is a prerequisite to adopt safe feces disposal practices and it is an important motivator to good hygiene behavior [38].

The current study also showed that improved water supply was associated with safe child feces disposal which is in line with other studies in Guinea [23] and Nigeria [37]. Accessibility of improved water is a strategy to reduce the susceptibility of children to diarrhea and other poor sanitation-induced health conditions. Hence, women who obtain water from unimproved sources are less motivated to practice safe fecal disposal [23].

## Limitation of the study

Since we used secondary data, we could not include important variables like knowledge and perception which have significant role for the outcome variable. Secondly, as the nature of cross-sectional study; the temporal relationship between the independent and outcome variable could not be established. The other limitation of this manuscript is; level-2 weight was not calculated. This may affect to know the effects of cluster-level variables on the outcomes (in our case safe child stool disposal). This can result in biased standard errors and parameters in analytical techniques that do not take the clustered nature of the data into account.

## Conclusion

Residence, mothers’ and partners’ level of education, occupational status of women, wealth index, family size, region, water source, toilet ownership and media exposure were factors associated with safe child feces disposal. Health promotion and behavioral intervention in this region should target on women/caregivers and their partners who are not educated, women from rural residence, with poor economic status, not employed, with>5 family members, cannot access improved water and women who had not media exposure to improve the practicability of safe child feces disposal.

## Supporting information

S1 File (DOCX)

## List of abbreviations

DHS: Demographic and Health Surveys
ICC: Interclass Correlation Coefficient
MOR: Median Odds Ratio
PCV: Proportional Change in Variance
SSA: sub-Saharan Africa
WHO: World Health Organization
