# Supplementary material for: Associated factors of safe child feces disposal in sub-Saharan Africa: Evidence from recent demographic and health surveys of 34 sub-Saharan countries
Source: PLoS One. 2023 Feb 9;18(2):e0281451. doi: 10.1371/journal.pone.0281451 (PMC9910663; doi:10.1371/journal.pone.0281451)
Supplement: S1 File — (DOCX) [file pone.0281451.s001.docx]

**Full model**

| **Variables** | **Null model** | **Model I AOR (95% CI)** | **Model II AOR (95% CI)** | **Model III AOR (95% CI)** |
| --- | --- | --- | --- | --- |
| Age of child(in month) |  |  |  |  |
| 0-11 |  | 1 | - | 1 |
| 12-23 |  | 0.91 (0.82-1.01) | - | 0.96(.82-1.02) |
| Age of mothers (years) |  |  | - |  |
| 15-24 |  | 1 | - | 1 |
| 25-34 |  | 1.02 (0.98-1.06) | - | 1.02(0.98-1.05) |
| 35 and above |  | 0.99 (0.95-1.07) | - | 0.99(0.94-1.04) |
| Education level of mothers |  |  | - |  |
| No education |  | 1 | - | 1 |
| Primary |  | 1.55 (1.46-1.61)* | - | 1.49(1.44-1.56)* |
| Secondary |  | 1.42 (1.53-1.49)* | - | 1.35(1.28-1.41)* |
| Higher |  | 0.88 (0.79-0.98)* | - | 0.85(0.76-0.94)* |
| Education level of Partner’s |  |  | - |  |
| No education |  | 1 | - | 1 |
| Primary |  | 1.22 (1.18-1.28)* | - | 1.23(1.18-1.28)* |
| Secondary |  | 1.19 (1.14-1.24)* | - | 1.16(1.11-1.22)* |
| Higher |  | 1.08 (0.99-1.17)* | - | 1.09(1.01-1.18)* |
| Respondents’ occupations |  |  | - |  |
| working |  | 1 | - | 1 |
| not working |  | 0.84 (0.882-0.87)* | - | 0.83(0.80-0.86)* |
| Number of under five children |  |  | - |  |
| ≤2 |  | 1.08(0.99-1.17) | - | 1.06(0.98-1.15) |
| 3-4 |  | 0.88(0.81-0.95) | - | 0.87(0.88-0.95)* |
| ≥5 |  | 1 | - | 1 |
| Family size |  |  | - |  |
| <5 |  | 1.07 (1.03-1.11)* | - |  |
| ≥5 |  | 1 | - |  |
| Wealth index |  |  | - |  |
| Poorest |  | 1 | - | 1 |
| Poorer |  | 1.35 (1.29-1.41)* | - | 1.34(1.28-1.41) )* |
| Middle |  | 1.56 (1.49-1.64)* | - | 1.51(1.44-1.48) )* |
| Richer |  | 1.97 (1.87-2.07)* | - | 1.78(1.69-1.88) )* |
| Richest |  | 2.67 (2.52-2.83)* | - | 2.17(2.01-2.31)* |
| Water source |  |  | - |  |
| Improved |  | 1.34 (1.29-1.39)* | - | 1.29(1.25-1.34)* |
| Un improved |  | 1 | - | 1 |
| Toilet ownerhip |  |  | - |  |
| Yes |  | 1 | - | 1 |
| No |  | 3.05 (2.96-3.15)* | - | 3.09(2.99-3.19)* |
| Media exposure |  |  | - |  |
| Yes |  | 1.23 (1.19-1.27)* | - | 1.19(1.15-1.24)* |
| No |  | 1 | - | 1 |
| Residence |  | - |  |  |
| Urban |  | - | 2.16 (2.09-2.23)* | 1.42(1.36-1.48)* |
| Rural |  | - | 1 | 1 |
| Region |  | - |  |  |
| East Africa |  | - | 1 | 1 |
| West Africa |  | - | 0.70 (0.67-0.73)* | 0.82(0.79-0.86)* |
| South Africa |  | - | 0.92 (0.88-0.96)* | 0.92(0.88-0.97)* |
| Central Africa |  | - | 0.69 (0.66-0.72)* | 0.84(0.79-0.87)* |
| Community-level variance | 0.53(0.47- 0.59) | 0.63(0.56- 0.71) | 0.49(0.43- 0.55)* | 0.50(0.44- 0.57) |
| ICC (%) | 13.88 % | 16.16% | 12.92% | 13.26% |
| MOR | 1.99 | 2.08 | 1.94 | 1.96 |
| PCV (%) | Ref. | 18.87% | 7.51% | 5.66% |
| Deviance | 356,185.76 | 346,401.12 | 308,537.76 | 306,855.18 |

*P≤0.05
